# GinJinn: An object‐detection pipeline for automated feature extraction from herbarium specimens

**DOI:** 10.1002/aps3.11351

**Published:** 2020-06-26

**Authors:** Tankred Ott, Christoph Palm, Robert Vogt, Christoph Oberprieler

**Affiliations:** ^1^ Evolutionary and Systematic Botany Group Institute of Plant Sciences University of Regensburg Universitätsstraße 31 D‐93053 Regensburg Germany; ^2^ Regensburg Medical Image Computing (ReMIC) Ostbayerische Technische Hochschule Regensburg (OTH Regensburg) Galgenbergstraße 32 D‐93053 Regensburg Germany; ^3^ Botanic Garden and Botanical Museum Berlin‐Dahlem Freie Universität Berlin Königin‐Luise‐Straße 6‐8 D‐14191 Berlin Germany

**Keywords:** deep learning, herbarium specimens, object detection, TensorFlow, visual recognition

## Abstract

**Premise:**

The generation of morphological data in evolutionary, taxonomic, and ecological studies of plants using herbarium material has traditionally been a labor‐intensive task. Recent progress in machine learning using deep artificial neural networks (deep learning) for image classification and object detection has facilitated the establishment of a pipeline for the automatic recognition and extraction of relevant structures in images of herbarium specimens.

**Methods and Results:**

We implemented an extendable pipeline based on state‐of‐the‐art deep‐learning object‐detection methods to collect leaf images from herbarium specimens of two species of the genus *Leucanthemum*. Using 183 specimens as the training data set, our pipeline extracted one or more intact leaves in 95% of the 61 test images.

**Conclusions:**

We establish GinJinn as a deep‐learning object‐detection tool for the automatic recognition and extraction of individual leaves or other structures from herbarium specimens. Our pipeline offers greater flexibility and a lower entrance barrier than previous image‐processing approaches based on hand‐crafted features.

Herbarium collections represent a rich treasure of plant specimens from around the world, providing the raw material for evolutionary, taxonomic, and ecological research. The increasing digitization of these natural history collections and their free availability allow scientists to tap into this treasure for systematic, historical, and phenological studies. The Global Biodiversity Information Facility (https://www.gbif.org) alone references herbaria containing over 30 million digitized plant specimens. Until recently, this source of data remained largely untouched due to the amount of manual labor required for the analysis of herbarium photographs.

Modern image‐processing methods, however, allow scientists to automate the analysis of digitized herbarium specimens (Corney et al., 2012a, b). In the past few years, progress in machine learning, especially the development of convolutional neural networks (CNNs), has made it possible to automatically identify the genus or species of herbarium specimens (Unger et al., 2016; Carranza‐Rojas et al., 2017), or even automatically extract qualitative information like leaf arrangement, form, and structure from digital images of preserved plants (Younis et al., 2018). Recently, Lorieul et al. (2019) showed that machine learning–based image classification can be used to detect the phenological state of herbarium specimens. An area of machine learning that has only very recently gained traction within the plant science community is the explicit object detection of plant structures such as leaves, flowers, or fruits in preserved specimens (Goëau et al., 2020; White et al., 2020).

Here, we introduce GinJinn, an object‐detection pipeline based on the TensorFlow (Abadi et al., 2016) object‐detection application programming interface (API) designed to make supervised deep‐learning object detection accessible for plant scientists. Its name relates to the “magical” [Jinn] extraction of herb(arium specimen)s [Gin] to detect morphological features/structures. GinJinn streamlines the process of moving from annotated images to a trained object‐detection model that can be exported and used for the automatic extraction of relevant structures of interest from newly acquired images of a particular study group. Thus, GinJinn allows scientists with little or no prior knowledge of machine learning to apply modern visual‐recognition tools and to incorporate object detection into their workflow by automatizing data‐mining processes that were previously largely manually performed.

We provide an automatic setup of projects for 47 different bounding‐box‐based object‐detection architectures together with the automatic download of pretrained models for 17 of them. While simplifying the process of model training and deployment, GinJinn still exposes the raw TensorFlow object‐detection API configuration files, which gives advanced users full access to all the architectural, preprocessing, and augmentation options provided by TensorFlow.

To show the efficacy of our pipeline, we used GinJinn to train and evaluate a model for the extraction of intact leaves from digitized herbarium specimens. From a technical point of view, the automatic extraction of leaves is an interesting problem, as software is already available for the automatic morphometry of isolated leaves (Corney et al., 2012a; Bonhomme et al., 2014; Chuanromanee et al., 2019) but the process of isolating the leaves themselves is not yet fully automatized (Corney et al., 2012b), especially not using modern machine learning techniques. From a biological point of view, leaf morphometry is an important tool for species delimitation and recognition, as well as for the reconstruction of historical climate conditions (Royer et al., 2005, 2008).

Here, we use two closely related *Leucanthemum* Mill. (Compositae, Anthemideae) species with different ploidy levels, namely the diploid *L. vulgare* Lam. and the tetraploid *L. ircutianum* DC., to demonstrate the application of leaf detection and extraction in a herbaceous plant group.

## METHODS AND RESULTS

### Software

GinJinn was originally developed as an internal tool for rapid iteration through deep‐learning model architectures to find adequate neural network models for the detection and extraction of intact leaves in digital images of herbarium specimens for subsequent morphometric analyses. It has since evolved into a general object‐detection pipeline for the setup, training, evaluation, and deployment of bounding‐box‐based object‐detection models with a focus on providing easy access to a high number of different model architectures with little manual work for the user, including the automated download of pretrained models if available. With GinJinn, we provide plant scientists a tool for applying modern machine learning–based visual recognition to their own data sets without requiring a thorough theoretical background in machine learning and proficiency in programming, which is generally necessary to apply and deploy deep‐learning object detection.

GinJinn is a Python 3 command‐line application for the management, training, and application of object‐detection models. In addition to the pipeline application, GinJinn contains several helper scripts that can be used separately from the main command line tool. GinJinn makes use of the free, open‐source deep‐learning framework TensorFlow (Abadi et al., 2016). Specifically, we are using the TensorFlow object‐detection API to access highly optimized training and evaluation pipelines and modern neural network architectures. The object‐detection models supported by GinJinn are bounding‐box prediction models; segmentation models are not yet implemented. This means that, based on sufficient training data where representative instances of the objects of interest are annotated with encompassing bounding boxes, the CNN learns to recreate those bounding boxes on the training data, and is also able to transfer the learned image‐to‐bounding‐box transformation to newly acquired, similar data (Girschick et al., 2013; Liu et al., 2015; see O’Shea and Nash [2015] for an introduction to CNNs). This allows the automatic recognition of structures of interest after the training of the neural network. In the context of herbarium specimens, those structures might be, for example, fruits, flowers, leaves, buds, or herbivore damage patterns. Structures extracted by GinJinn may be subsequently subjected to different downstream analyses aiming to quantify their shape, color, or texture; count different structure classes (number of buds vs. number of flowers vs. number of fruits in phenological studies); or quantify their positions relative to each other on the surveyed herbarium specimen (coordinates of members of a predefined structure class).

The two different meta‐architectures of bounding‐box‐predicting models that are supported by GinJinn are Regions with CNNs (R‐CNNs; Girschick et al., 2013) and Single Shot Multibox Detectors (SSDs; Liu et al., 2015). R‐CNNs basically employ a two‐step procedure of first predicting regions of interest, so‐called region proposals, and subsequently classifying the regions of interest (Girschick et al., 2013; Girschick, 2015; Ren et al., 2015). In contrast, SSDs combine both steps in a single neural network architecture (Liu et al., 2015). While SSDs are more modern and allow faster prediction of bounding boxes, a recent benchmarking study by Zhao et al. (2018) showed that R‐CNNs achieve similar or better accuracies. The even more recently developed class of bounding‐box predicting models, You Only Look Once (YOLO) (Redmon et al., 2015), is intentionally not supported by GinJinn, because these models focus on prediction speed by sacrificing accuracy (Zhao et al., 2018), which is not necessary for the extraction of structures from static images of preserved plants.

Although GinJinn makes heavy use of the TensorFlow object‐detection API, it is not merely a wrapper to ease the use of the API. GinJinn provides additional tools for data preprocessing, setting up a standardized project structure, downloading pretrained models (if available), simple model exporting, and using the trained network for the extraction of bounding boxes from newly acquired data, which is a functionality not supported out‐of‐the‐box by the TensorFlow object‐detection API. While providing this additional functionality, we ensured that the intermediary and output files were kept compatible with TensorFlow to allow advanced users to seamlessly access the more advanced features of the TensorFlow object‐detection API without having to leave the framework provided by GinJinn. Hence, for users who are new to the field of machine learning–based object detection, the pipeline can act as a gentle introduction and allow them to iteratively try out more advanced functionalities of modern deep‐learning object detection. Additionally, the interoperability with TensorFlow allows GinJinn users to monitor the training and evaluation of their models live with the TensorBoard (Abadi et al., 2016) tool.

**FIGURE 1:**
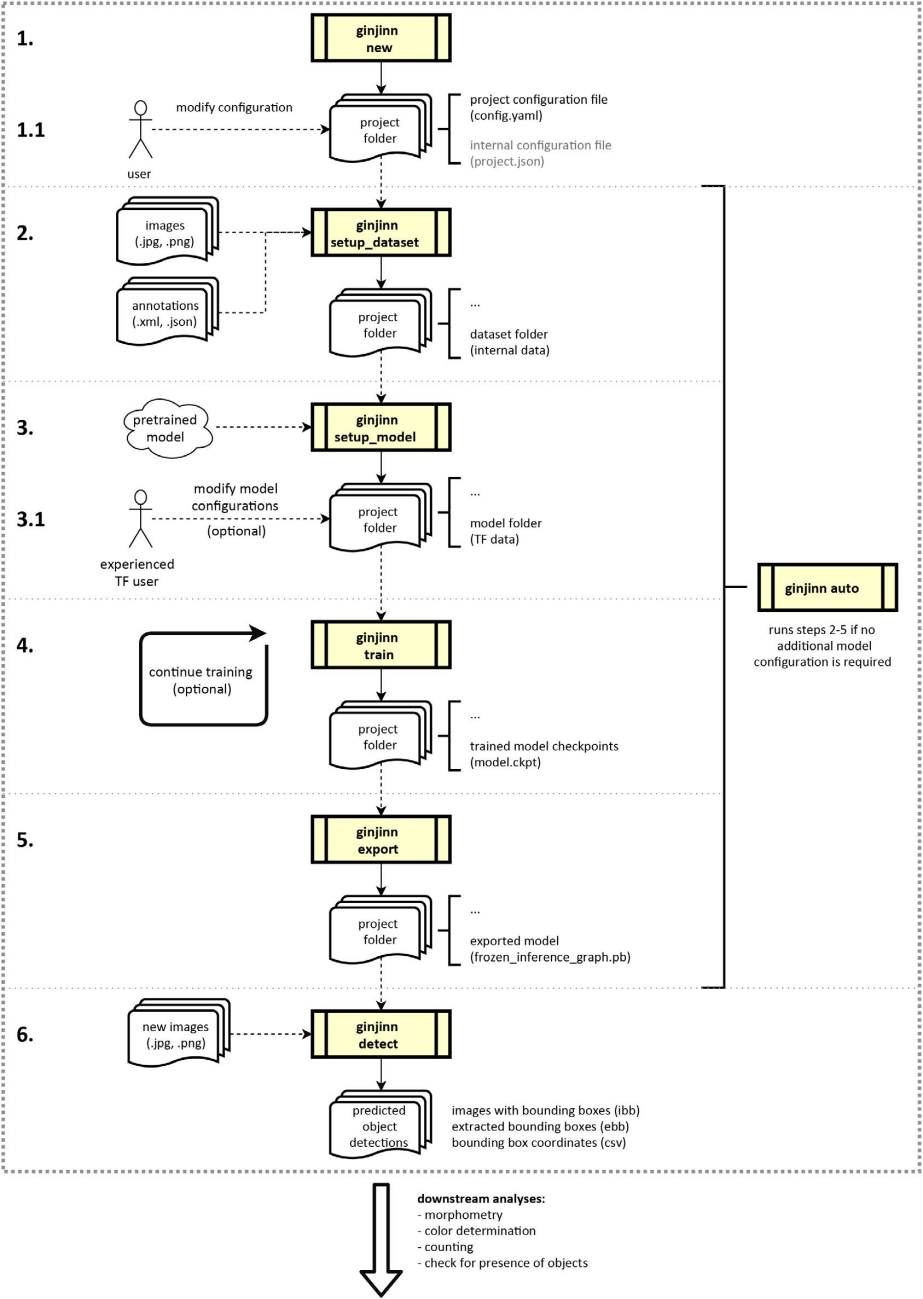
Flow diagram of the six GinJinn pipeline steps. A project folder is generated using *ginjinn new* (1) and the configuration file is modified depending on the user’s needs (1.1). The preparation (2), processing (3), training (4), and export (5) steps are executed sequentially with specific GinJinn commands (*setup*_*dataset*, *setup*_*model*, *train*, and *export*, respectively), or alternatively at once with the single *ginjinn auto* command. When not using *ginjinn auto*, the user can modify intermediary TensorFlow configuration files (3.1) for additional control over the model parameters and augmentation options. The trained and exported model can be used for inference of bounding boxes on new data using *ginjinn detect*. GinJinn commands are indicated by the yellow process boxes. Data inputs and outputs are illustrated with solid and dashed arrows, respectively. After bounding box detection, the extracted structures of interest can be supplied to other tools for downstream analyses.

The GinJinn pipeline consists of six steps (Fig. 1): (1) The generation of a project directory including a project configuration template file. The project configuration file is the place for the user to set data paths and select parameters for the subsequent pipeline steps. (2) The conversion of the data to an internal format and splitting of the data into training and test data sets. (3) Model preparation, which includes the setup of the model and the automatic download of pretrained models (if desired and available). Additionally, this step generates the TensorFlow model configuration file. Advanced users can modify this file to influence image preprocessing, as well as the training and evaluation of the model. (4) Simultaneous model training and evaluation. Model checkpoints are automatically saved. During this step, progress can be monitored via TensorBoard or the console output. (5) Model export, in which the user can select one saved model checkpoint for export. (6) The use of exported models for the extraction of structures from newly acquired images via an additional GinJinn command.

GinJinn accepts JPEG (.jpg, .jpeg) and PNG (.png) images with corresponding annotations in PASCAL Visual Object Classes Challenge (VOC; Everingham et al., 2010) XML format or VGG Image Annotator (Abhishek and Zisserman, 2019) JSON format for the training and evaluation of the CNNs. PNG images can be supplied only without the alpha channel (transparency). Like the exported models, the intermediary outputs are also compatible with standard TensorFlow object‐detection workflows. The prediction of bounding boxes on newly obtained image data can be performed based on both JPEG and PNG formats. The output of the prediction is available as class‐wise images with bounding boxes for visual inspection, cropped bounding boxes, or bounding‐box coordinates in CSV (.csv) format. The output image formats are PNG or JPEG, depending on the format of the respective input images.

We have tested GinJinn on Windows 10 (Microsoft Corporation, Redmond, Washington, USA), Debian (https://www.debian.org/), and Ubuntu (https://ubuntu.com/). The pipeline requires an installation of Python 3.6 (van Rossum and Drake, 2009) and a corresponding TensorFlow or TensorFlow–graphics processing unit (GPU) version. The latter version is recommended due to the speedup in training, evaluation, and inference time compared to the CPU version, but requires the installation of proprietary NVIDIA GPU drivers and toolkits. Apart from the computation time, both versions are equivalent. Detailed installation instructions can be found in the manual. GinJinn has been released open source under the MIT license. The source code, including the installation instructions, is available at https://github.com/AGOberprieler/ginjinn.

### Example application: *Leucanthemum* leaves

As an example of the application of GinJinn**,** we present the recognition and extraction of intact leaves from digital images of preserved herbarium specimens of two species of *Leucanthemum* (ox‐eye daisies; Compositae, Anthemideae) with different ploidy levels, namely the diploid *L. vulgare* and the tetraploid *L. ircutianum*. One important morphological character for the differentiation of those two species is the shape of the basal and middle cauline leaves (Wagenitz, 1977; Vogt, 1991).

The automated recognition of intact leaves on herbarium specimens, especially for plants with a high variability in leaf shape—as is the case for *Leucanthemum*—can be considered a complex task, because the occurrence of intact leaves in relation to the occurrence of non‐intact leaves is rare. This high abundance of damaged leaves is caused by factors such as herbivore damage, shearing while handling the vouchers, and most prominently by the dry pressing process, where leaves are often unintendedly folded. Additionally, the difference between damaged and intact leaves can be very small, making it hard to clearly differentiate between the two cases, even for human observers. We defined as intact those leaves that were completely visible, non‐overlapping, non‐folded, and not damaged by herbivores. For cauline leaves, special care was taken to ensure that the leaf base was visible, as this is an important character for the distinction between the two *Leucanthemum* species (Wagenitz, 1977; Vogt, 1991). Damaged, overlapping, or folded leaves were not annotated. Accordingly, the detection of intact leaves in this study is posed as a single‐class bounding‐box detection problem. We have refrained from the subsequent downstream analyses of extracted structures (here: leaves) because these analyses of taxonomically relevant features such as the leaf outline, degree of dissection, color, or texture could be easily accomplished with existing software such as MASS (Chuanromanee et al., 2019) or Momocs (Bonhomme et al., 2014).

For the present example application of GinJinn, we used a data set consisting of 286 JPEG images of preserved plant herbarium specimens provided by the herbarium of the Botanic Garden and Botanical Museum Berlin‐Dahlem (B), Berlin, Germany. The images were annotated using the free open‐source tool LabelImg version 1.8.1 (https://github.com/tzutalin/labelImg), resulting in a total of 889 annotated intact leaves in 243 images of herbarium specimens. For the 43 remaining images, no intact leaves were present. GinJinn was used to split the data into training and test data sets for model evaluation by randomly sampling 25% of the images into the test data set.

A model architecture consisting of a Faster R‐CNN (Ren et al., 2015) meta‐architecture and Inception‐ResNet version 2 (Szegedy et al., 2016) as the feature extractor was selected. To speed up the training process, we applied so‐called transfer learning by starting the training from a model that was pretrained on the Common Objects in Context (COCO) data set (Lin et al., 2014) provided by the TensorFlow object‐detection API. The model was trained for 12,000 generations with a batch size of 1.

**FIGURE 2:**
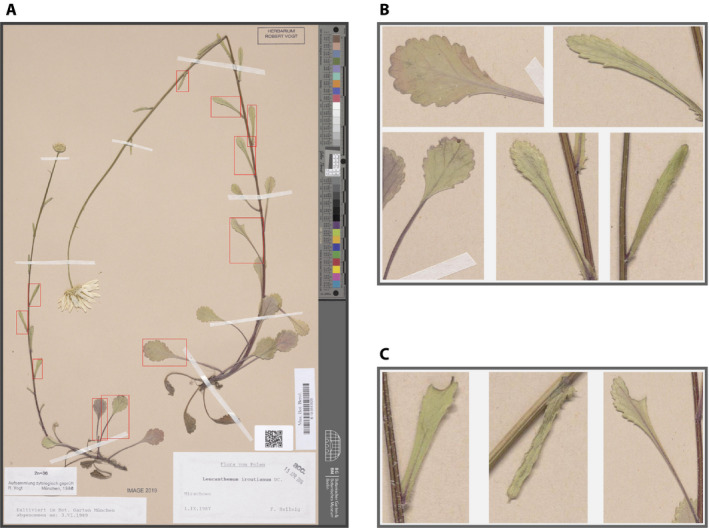
(A) Output type ‘ibb’ (image with bounding boxes) showing class‐wise predicted bounding boxes of leaves with a score of 0.5 or higher drawn on the original image of a herbarium specimen. The score can be interpreted as a probability that the content of the bounding box belongs to a certain object class (in this case, a leaf). (B) Output type ‘ebb’ (extracted bounding boxes with a padding of 25 pixels) for selected true positive examples of the detected leaves shown in A. (C) Output type ‘ebb’ for selected false positive examples of the leaves shown in A.

The evaluation was performed according to the PASCAL VOC challenge evaluation metrics (Everingham et al., 2010). The model achieved a mean average precision (mAP) of 0.49 at an intersection over the union (IoU) of 50%. We were able to successfully detect the presence of one or more intact leaves in 95% of the 61 test images, for which the presence of an intact leaf was manually determined a priori. Figure 2A shows the resulting predicted bounding boxes on a test image, with Fig. 2B and C depicting true positive and false positive leaf detection, respectively. The results shown in Fig. 2B could subsequently be used for morphometric analyses with tools such as MASS (Chuanromanee et al., 2019) or Momocs (Bonhomme et al., 2014), for example. Our results indicate the applicability of training a deep‐learning model for the detection of objects in preserved plant specimens that can potentially assist or even automatize the extraction of leaves from herbarium images or assist further annotations, even with a relatively small data set of only 243 images.

The software manual hosted at the GinJinn GitHub repository contains a dedicated section for the reproduction of these results. This section should also be considered a tutorial for new users.

## CONCLUSIONS

With GinJinn, we are introducing a new software tool that allows plant scientists to tap into modern deep‐learning‐based visual recognition for the exploration and exploitation of the rich treasure that digitized herbarium specimens in collections all over the globe represent. Here, we have shown that our pipeline is able to automatically extract intact leaves from herbarium specimen images for subsequent downstream analyses. This provides the potential to speed up and automatize previously work‐intensive manual workflows, and can grant scientists access to huge amounts of morphological data for morphometric and phenological studies using herbarium specimens.

Previous work in the area of visual recognition of preserved plant materials used hand‐crafted features for trait and structure extraction (Corney et al., 2012b; Henries and Tashakkori, 2012; Unger et al., 2016), focused on classification instead of object detection (Jin et al., 2015; Munisami et al., 2015; Carranza‐Rojas et al., 2017; Younis et al., 2018; Lorieul et al., 2019), or tried to directly extract characters from images without using explicit object‐detection techniques (Ubbens and Stavness, 2017; Younis et al., 2018). GinJinn, in contrast, is a tool specifically developed for the extraction of structures such as leaves, flowers, buds, or fruits from digitized herbarium specimens. As such, GinJinn can be used to generate inputs for downstream analyses with existing tools, for example, the recently released MASS (Chuanromanee et al., 2019) software for morphometric analyses. A tool somewhat similar to GinJinn, LeafMachine (Weaver et al., 2020), is also newly available for the extraction of leaves from digital images of preserved plants. Whereas LeafMachine is designed to extract leaves via semantic segmentation, our pipeline can be used to extract instances of any kind of structure that it is trained for via bounding‐box object detection. Furthermore, all dependencies of GinJinn are free and open source, while LeafMachine depends on the proprietary MATLAB (MathWorks, Natick, Massachusetts, USA) environment. However, if a pixel‐wise segmentation is required instead of cropped leaves, LeafMachine might be the better‐suited tool. Another possibility would be to use both tools: GinJinn to first reduce the complexity of the problem via bounding‐box cropping and a subsequent pixel‐perfect extraction of leaf silhouettes based on the cropped leaves using LeafMachine.

When compared to the usage of the TensorFlow object‐detection API directly, our pipeline adds the additional features of project setup, data preparation, automatic download of pretrained models, and an easy‐to‐use inference routine with outputs fitted to the plant science community. Additionally, GinJinn can be used by scientists without proficiency in Python programming and generally does not require any knowledge about the architecture of TensorFlow and the TensorFlow object‐detection API.

By designing the pipeline with ease of use in mind, it was necessary to reduce the feature set that is presented to the user when compared to TensorFlow. This drawback is partly mitigated by exposing the raw TensorFlow configuration and the run and export scripts in the project folders generated by GinJinn in such a way that advanced users can modify those files directly without leaving the framework of the pipeline.

As a machine learning–based tool, the performance of object‐detection models trained using GinJinn is highly dependent on the quality of the available training data. This limitation, however, applies to all machine learning–based modeling. Care must be taken to ensure the data used for training the object‐detection models resembles the expected test data. A problem, for example, are strong differences in lighting conditions or the angle from which images were taken between the training and test data. This limitation is partially mitigated using the built‐in augmentation options, which introduce small perturbations into the training images to make the model more resistant against that type of variability.

A temporary technical restriction is that only bounding‐box‐predicting models are available, even though models for semantic and instance segmentation are also supported by the TensorFlow object‐detection API. However, in future versions, those segmentation models will be made available through the GinJinn interface. Another goal for the next version of the application is to provide the configuration of additional data augmentation options. The long‐term aim is the integration of the PyTorch (Paszke et al., 2017) framework as an alternative to TensorFlow, which would introduce a higher amount of available architectures and an easier setup of GPU acceleration for GinJinn.

We present GinJinn as a deep‐learning object‐detection tool for the automatic recognition and extraction of structures such as leaves or flowers from herbarium specimens. We showed that GinJinn can be applied to successfully extract intact leaves from images of herbarized *Leucanthemum* individuals, while offering greater flexibility and a lower barrier to entry compared with previous image‐processing approaches based on hand‐crafted features.

## AUTHOR CONTRIBUTIONS

T.O., C.P., and C.O. conceived the present study. R.V. produced the scans of the preserved specimens. T.O. programmed the software. C.P. consulted the exemplary data analysis. A first draft of the paper was written by T.O. with input from C.P., R.V., and C.O.

## Data Availability

Source code and manual are hosted at https://github.com/AGOberprieler/ginjinn. The annotated image data that were used for evaluation of the method are hosted by the German Federation for Biological Data (GFBio; https://data.bgbm.org/dataset/gfbio/0033/).
